# Pattern of Uveitis in a Tertiary Hospital in North Jordan and the Impact of Behcet's Disease

**DOI:** 10.1155/2023/2076728

**Published:** 2023-08-08

**Authors:** Khaldoon M. Alawneh, Omar A. Saleh, Mahmoud M. Smadi, Fatima Kamel Ababneh, Ikhlas Hamed Ali Mahmoud, Areje M. Smadi, Diala Alawneh

**Affiliations:** ^1^Faculty of Medicine, Jordan University of Science and Technology, Irbid, Jordan; ^2^Department of Internal Medicine, King Abdullah University Hospital, Irbid, Jordan; ^3^Department of Ophthalmology, Jordan University of Science and Technology, Irbid, Jordan; ^4^Department of Mathematics and Statistics, Jordan University of Science and Technology, Irbid, Jordan; ^5^Jordan University of Science and Technology, Irbid, Jordan; ^6^Division of Rheumatology, University of Illinois at Chicago, Chicago, IL, USA

## Abstract

**Aim:**

The purpose of this study is to assess the prevalence of autoimmune-mediated uveitis in relation to other diseases and to describe the clinical patterns of uveitis in a single tertiary hospital in north Jordan.

**Methods:**

A cross-sectional retrospective review was performed. We included all patients diagnosed with uveitis in King Abdullah University Hospital (KAUH) ophthalmology clinic and/or patients referred to KAUH rheumatology clinics for evaluation of suspected autoimmune mediated uveitis or for difficult to treat uveitis. This included patients from January 2015 to January 2019. Data collected about patients' age, sex, anatomical location of the disease, etiology, treatment, complications, and outcomes on vision loss were analyzed.

**Results:**

A total of 221 patients were included in our study. The mean (±SD) age was 36 (±16) years. A total of 111 patients were female and 110 were male with a ratio of 1 : 1. Noninfectious uveitis was found to be more common than infectious uveitis (95% vs. 5% respectively). Autoimmune-mediated uveitis accounted for 40% of the total cases. The most common causes of autoimmune-mediated uveitis included Behcet's disease (*n* = 41, 19%) and seronegative spondyloarthropathy (*n* = 29, 13%). The majority of patients (*n* = 207, 94%) were treated with ophthalmic eye drops, cDMARDs (*n* = 101, 46%), biologics (*n* = 33, 15%), and colchicine (*n* = 30, 14%).

**Conclusion:**

Autoimmune-mediated uveitis, most commonly associated with Behcet's disease and seronegative spondyloarthropathy, represents a high percentage of uveitis in our area. This implies the need for a high index of suspicion at the time of evaluation.

## 1. Introduction

Uveitis is an intraocular inflammatory disease of the uveal tissues that is categorized based on anatomical involvement into anterior, intermediate, posterior, and panuveitis. It can attack non-uveal tissues such as the retina, optic nerve, and sclera. It is a vision threatening disease that predominately affects patients during their working years, causing an increase in medical costs and rates of disability compared to age matched peers [1]. Uveitis can be idiopathic or secondary to trauma, infection, or autoimmune diseases such as spondyloarthropathy, Behcet's disease, and sarcoidosis.

The prevalence of uveitis varies in each autoimmune disease. For example, the prevalence is as high as 33% in seronegative spondyloarthropathy compared to 2.3% in psoriatic arthritis [2]. In addition, the prevalence of autoimmune diseases and their clinical presentations vary according to geography. For example, the prevalence of Behcet's disease is 4.9/100,000 in southern Sweden [3] compared to 20/100,000 in rural western Turkey [4].

Jordan has a high prevalence of Behcet's disease, which is estimated to be 660/100,000 in northern Jordan [5]. Studies in Jordan have shown that up to 65% of patients with Behcet's develop ocular manifestations [6, 7].

Given the limited data on uveitis in the surrounding area in general [8–15] and Jordan in particular, we aim to assess the prevalence of uveitis and its association with autoimmune diseases in Jordan.

## 2. Materials and Methods

A cross-sectional retrospective study including all patients diagnosed with uveitis at King Abdullah University Hospital (KAUH) ophthalmology clinics and/or referred to rheumatology clinics with a diagnosis of uveitis was performed. This study was performed according to strengthening the reporting of observational studies in epidemiology (STROBE) guidelines [16]. The Institutional Review Board and Research Ethics Committee approved the study.

Patients' records were reviewed and data regarding age of diagnosis, gender, details of ocular examination (uveal and non-uveal parts involved and unilateral or bilateral eye involvement), etiology, treatment, and complications were collected.

Intraocular inflammation that was not associated with a specific underlying disease or ocular entity was categorized as “idiopathic uveitis.” Uveitis cases were divided into anterior, intermediate, posterior, or panuveitis as per standardization of uveitis (SUN) anatomic classification [17].

Normally distributed continuous variables were reported as means ± standard deviation (SD) and categorical variables were reported as counts and percentages. All statistical analyses were performed using the statistical package SPSS 21.0 (SPSS Inc. Chicago, IL).

## 3. Results

**Figure 1:**
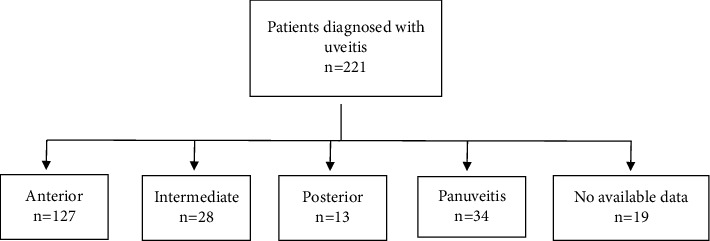
Flowchart of the population selection process.

A total of 221 patients with uveitis were diagnosed at or referred to KAUH from January 2015 to January 2019; 110 were female (49.8%) and 111 were male (50.2%) (Figure 1). The mean (±SD) age was 36 (±16) years with ages ranging from 3 to 78 years. Information about smoking was only reported in 56 patients with a total of 38 (67.9%) nonsmokers and 18 (32.1%) smokers.

Noninfectious uveitis (*n* = 211, 95.5%) was more common than infectious uveitis (*n* = 10, 4.5%). The most common cause of noninfectious uveitis was idiopathic (*n* = 101, 45.7%) followed by autoimmune-mediated uveitis (*n* = 88, 39.8%).

Amongst autoimmune-mediated uveitis, the etiology varied with 41 (18.6%) cases of uveitis associated with Behcet's disease, 29 (13.2%) with seronegative spondyloarthropathy, 11 (5%) with juvenile idiopathic arthritis (JIA), 4 with sarcoidosis (1.8%), 3 (1.4%) with rheumatoid arthritis (RA), 2 (0.9%) with systemic lupus erythematosus (SLE), and 1 (0.5%) with Sjogren's disease. The 29 cases of seronegative spondyloarthropathy included 9 cases of undifferentiated spondyloarthropathy, 7 cases of psoriatic arthritis, 6 cases of ankylosing spondylitis, 3 cases of inflammatory bowel disease (IBD) associated arthritis, 2 cases of reactive arthritis, and 2 cases of juvenile spondyloarthropathy. Three patients had both Behcet's disease and psoriasis.

In addition, there were 7 (3.2%) cases of Vogt-Koyanagi-Harada (VKH), 5 (2.3%) cases of Fuchs heterochromic iridocyclitis, 1 (0.5%) case of familial mediterranean fever (FMF), 1 (0.5%) case of agammaglobulinemia, 1 (0.5%) case of tubulointerstitial nephritis and uveitis syndrome (TINU), and 1 (0.5%) case of Posner-Schlossman syndrome.

Infectious uveitis etiologies included 5 cases of herpetic kerato-uveitis, 3 cases of toxoplasmosis, 1 case of tuberculosis, and 1 case of infective endocarditis. A total of 4 cases developed post-cataract surgery, 2 post-trauma, and one due to a foreign body.

Antinuclear antibody (ANA) testing was performed in a total of 142 (64.3%) patients; of which 34 (23.9%) tested positive and 108 (76.1%) negative. In addition, human leukocyte antigen B27 (HLA-B27) testing was performed in a total of 45 (20.4%) patients; of which 12 (26.7%) patients tested positive and 33 (73.3%) negative.

Unilateral eye involvement was the most common presentation (*n* = 115, 52.0%), with 66 (34.2%) patients having left eye disease and 49 (25.4%) having right eye disease. Bilateral eye involvement was present in 78 (40.4%) patients. Data were missing in 28 patients.

Uveitis was also categorized based on anatomical involvement into anterior, intermediate, posterior, and panuveitis. A total of 127 (55.2%) patients had anterior uveitis, 28 (12.7%) intermediate, 13 (3.6%) posterior, and 34 (16.7%) panuveitis. Data were missing for 19 patients. The etiologies of uveitis in relation to anatomical classification of the disease are presented in Table 1. Smoking was associated with increased odds of posterior uveitis only (OR = 3.5, 95% CI: 2.3–5.4, *p*=0.029).

Treatment of uveitis varied based on etiology. However, most patients (*n* = 207, 93.7%) received topical steroids. Posterior subtenon triamcinolone acetonide injections were performed in 24 (10.9%) patients, 3 (1.4%) patients required vitrectomy, and 2 (0.9%) had intraocular steroid implants inserted.

Systemic steroids were used in 78 (35.3%) patients, while colchicine was used in 30 (13.6%) patients. Almost half (*n* = 101, 45.7%) of our patient population was treated with conventional disease modifying antirheumatic drugs (cDMARDs), either with single or combination therapy. This included 57 patients treated with azathioprine, 30 with methotrexate, 11 with mycophenolate mofetil, 7 with cyclosporine, 6 with sulfasalazine, and 4 with hydroxychloroquine. A total of 37 (18.4%) patients were treated with biologic agents (either switched for effectiveness or unavailability of other drugs), including infliximab (*n* = 23), adalimumab (*n* = 12), etanercept (*n* = 1), and tocilizumab (*n* = 1).

Complications included cataract (*n* = 39, 17.6%), glaucoma (*n* = 36, 16.3%), epiretinal membrane (*n* = 17, 7.7%), band keratopathy (*n* = 6, 2.7%), macular edema (*n* = 5, 2.3%), synechia (*n* = 3, 1.4%), retinal scarring (*n* = 3, 1.4%), retinal vein occlusion (*n* = 3, 1.4%), macular dragging (*n* = 1, 0.5%), retinal hole (*n* = 1, 0.5%), squint (*n* = 1, 0.5%), nystagmus (1 (0.5%), macular scar (*n* = 1, 0.5%), and retinal detachment (*n* = 1, 0.5%).

**Figure 2:**
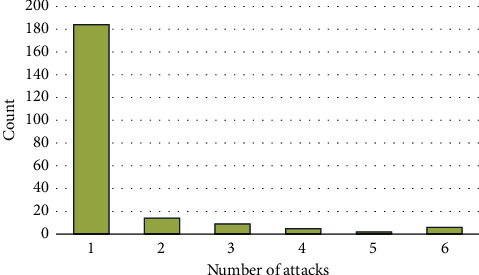
Distribution of number of attacks.

**Figure 3:**
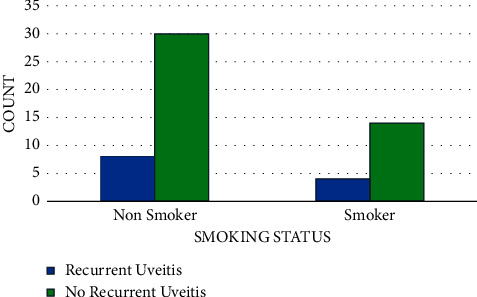
Relation of smoking and recurrence of uveitis.

A total of 36 (16.4%) patients had recurrent disease (Figure 2). There were 6/11 (54.5%) JIA patients with a recurrence of disease (*p*=0.003). In Behcet's disease, 8/41 (19.5%) patients had recurrence of their uveitis (*p*=0.639). While in VKH patients, 3/7 (42.9%) had reoccurrence (*p*=0.088). Patients with JIA had significantly higher rates of recurrence compared to other disease processes. In addition, smoking was not found to have statistical significance in relation to recurrence rate (*p*=0.92) (Figure 3).

## 4. Discussion

Uveitis is a vision threatening disease that has serious implications for patients and for the socioeconomic status of a community. It is responsible for 20% of legal blindness [13]. Herein, we reviewed and described the patterns of uveitis in north Jordan. Similar to the general population, the patients in our study were relatively young with a mean age of 36-years. Males and females were equally affected [14, 18–25].

Anterior uveitis was the most common form of uveitis, involving roughly half of our population similar to that in other studies [26–29]. Unilateral eye involvement was more common than bilateral, with left eye disease being slightly more prevalent than right.

Idiopathic uveitis was the most common etiology of uveitis in our study, which is higher than that reported in other Middle Eastern and Asian countries with a prevalence of 45.7% vs. 24.9–36%, respectively, [14, 30–32]. However, one review including 15,221 patients across several countries showed similar results with 44.6% of cases attributed to idiopathic uveitis [33].

Autoimmune-mediated uveitis is typically the second most common cause of uveitis with almost 40% of the population receiving this diagnosis. This highlights the importance of evaluating patients with uveitis for a systemic autoimmune disease, as many times this can be the presenting symptom. In our region, Behcet's disease and seronegative spondyloarthropathies were the most frequently encountered. They included almost 80% of autoimmune mediated uveitis cases.

Posterior or panuveitis are the typical presentations associated with Behcet's disease, while anterior and intermediate uveitis are considered rare [34]. Interestingly, in our group, anterior uveitis was the most common followed by panuveitis. However, three patients who had anterior uveitis had concomitant psoriasis in addition to their Behcet's disease, which likely explains this difference.

There were a total of 18.6% patients with Behcet's uveitis, which is almost three times that found in Japan. This is expected as Behcet's disease is not common in that region [28]. Although Behcet's disease is most prevalent in the Middle Eastern region, studies from surrounding areas such as Saudi Arabia, Iraq, and Iran showed a lower number of cases compared to north Jordan [11–14]. However, multiple studies from Turkey show a significantly higher prevalence ranging from 26 to 32% [25, 34].

Seronegative spondyloarthropathy was the cause of uveitis in 13.2% of our population. This is higher than that in several studies across the globe [13, 20, 25, 26, 29, 34]. This included HLA-B27 positive uveitis despite only a fifth of our patients having this test performed. This leads us to believe there may be a higher prevalence in our area than that known. Most studies did not mention other seronegative subtypes such as psoriatic arthritis and IBD associated arthritis, which led to a limitation when comparing results.

Only 3.2% of cases were due to VKH associated uveitis, which is higher than that found in a tertiary Iranian eye center with a very low prevalence of 0.69% [35]. However, it is lower than most other studies where results ranged between 8.7 and 15.9% [14, 36, 37]. We believe this may be due to our smaller sized sample. We had one case of uveitis in a patient with FMF, which we believe was coincidental in nature as FMF which is not known to cause uveitis.

Studies have shown an association between smoking and uveitis [38, 39]. The Pacific ocular inflammation study, which included 224 patients, showed a 2-fold higher odds of uveitis in smokers [38]. One study showed smoking was associated with all types of uveitis, but more specifically in those with intermediate or panuveitis [39]. Although we had limited data regarding smoking, there was a significant association between smoking and the development of posterior uveitis.

The majority of patients received topical steroids as a part of their uveitis treatment. Almost half, required treatment with cDMARDs and 15% received biologics. One patient received etanercept; however, this was not used to treat his uveitis as etanercept is not effective for these cases. This patient had been on treatment for his seronegative spondyloarthropathy prior to the development of his eye disease. His uveitis was managed with topical steroids. In addition, colchicine was used as an add on therapy in some cases.

We acknowledge the limitations of our study related to its retrospective nature where confounders are not accounted for. Second, we included patients that were seen at a rheumatology clinic. This might introduce a selection bias, as these patients have a higher pretest probability of autoimmune disease. Furthermore, this was a single institution study. This may limit the generalizability of our results. Lastly, this was purely a descriptive study which limited the ability to compare presentations and outcomes of different uveitis etiologies.

However, this remains the first study to describe uveitis patterns in adults in Jordan. Here, we highlight the high prevalence and the significant impact of autoimmune diseases such as Behcet's and seronegative spondyloarthropathies on uveitis in our region. Given the high prevalence of autoimmune uveitis in our cohort, a high index of suspicion is required when evaluating patients with uveitis. In our study, only a limited number of patients were tested for HLA-B27 and ANA as they were performed based on clinical suspicion. Though these autoimmune diseases are typically clinically diagnosed, our data suggests that there might be a role for routine HLA-B27 and ANA testing in all patients with idiopathic uveitis in Jordan. This might apply even in the absence of typical systemic manifestations of autoimmune disease, as uveitis may be the presenting symptom.

## 5. Conclusion

The prompt diagnosis and treatment of uveitis is necessary to avoid devastating outcomes such as blindness. Uveitis associated with autoimmune diseases, such as Behcet's and seronegative spondyloarthropathy, is common, especially in our region. It is an important differential to consider. Patients were most commonly treated with topical corticosteroids, cDMARDS, biologics, and colchicine.

## Figures and Tables

**Table 1:** Etiology of uveitis in relation to its type.

Uveitis etiology	Total (*n* = 221)^*∗*^	Anterior (*n* = 127)^*∗∗∗*^	Intermediate (*n* = 28)	Posterior (*n* = 13)	Panuveitis (*n* = 34)
	*n* (%)	*n* (%)	*n* (%)	*n* (%)	*n* (%)
Idiopathic	101 (45.7)	61 (48)	20 (71.42)	5 (38.46)	12 (35.29)
*Autoimmune-mediated*	88 (39.8)	50 (39.4)	7 (25)	4 (30.77)	13 (38.23)
Behcet's disease	41 (18.6)	16 (12.59)^*∗∗*^	3 (10.71)	3 (23.07)	10 (29.41)
Spondyloarthropathy	29 (13.2)	24 (18.89)	1 (3.57)	1 (7.69)	1 (2.94)
JIA	11 (5)	10 (7.87)	0	0	1 (2.94)
RA	3 (1.4)	2 (1.57)	0	0	0
Sarcoidosis	4 (1.8)	1 (0.78)	1 (3.57)	0	1 (2.94)
SLE	2 (0.9)	0	1 (3.57)	0	0
Sjogren's disease	1 (0.5)	0	1 (3.57)	0	0
VKH	7 (3.2)	1 (0.78)	0	1 (7.69)	6 (17.65)
Fuchs heterochromic iridocyclitis	5 (2.3)	4 (3.14)	0	0	0
FMF	1 (0.5)	1 (0.78)	0	0	0
Agammaglobinemia	1 (0.5)	1 (0.78)	0	0	0
TINU	1 (0.5)	0	0	0	0
Posner-Schlossman syndrome	1 (0.5)	1 (0.78)	0	0	0
Infectious uveitis	10 (4.52)	4 (3.14)	0	3 (23.07)	2 (5.88)
Herpetic kerato-uveitis	5 (2.3)	4 (3.14)	0	0	0
Toxoplasmosis	3 (1.4)	0	0	3 (23.07)	0
Tuberculosis	1 (0.5)	0	0	0	1 (2.94)
Infective endocarditis	1 (0.5)	0	0	0	1 (2.94)
Postcataract surgery	4 (1.8)	2 (1.57)	1 (3.57)	0	1 (2.94)
Post-trauma	2 (0.9)	1 (0.78)	0	0	0
Foreign body	1 (0.5)	1 (0.78)	0	0	0

Note: ^*∗*^Total patients = 221 and total known chamber = 194. ^*∗∗*^3 patients had Behcet's disease and psoriasis. ^*∗∗∗*^2 patients had anterior and intermediate +6 patients had anterior and posterior. No data regarding chamber involved were available for 19 patients. FMF: familial mediterranean fever, TINU: of tubulointerstitial nephritis and uveitis syndrome, RA: rheumatoid arthritis, SLE: systemic lupus erythematosus, JIA: juvenile inflammatory arthritis, VKH: Vogt-Koyanagi-Harada.

## Data Availability

The datasets used during the current study are available from the corresponding author on request.

## References

[1] Thorne J. E., Skup M., Tundia N. (2016). Direct and indirect resource use, healthcare costs and work force absence in patients with non-infectious intermediate, posterior or panuveitis. *Acta Ophthalmologica*.

[2] Zeboulon N., Dougados M., Gossec L. (2008). Prevalence and characteristics of uveitis in the spondyloarthropathies: a systematic literature review. *Annals of the Rheumatic Diseases*.

[3] Mohammad A., Mandl T., Sturfelt G., Segelmark M. (2013). Incidence, prevalence and clinical characteristics of Behcet’s disease in southern Sweden. *Rheumatology*.

[4] Cakir N., Dervis E., Benian O. (2004). Prevalence of behçet’s disease in rural western Turkey: a preliminary report. *Clinical & Experimental Rheumatology*.

[5] Madanat W. Y., Alawneh K. M., Smadi M. M. (2017). The prevalence of behçet’s disease in the north of Jordan: a hospital-based epidemiological survey. *Clinical & Experimental Rheumatology*.

[6] Al-Aboosi M. M., Al Salem M., Saadeh A. (1996). BEHÇET’S disease: clinical study of JORDANIAN patients. *International Journal of Dermatology*.

[7] Abu-Ameerh M. A., Mohammed S. F., Mohammad M. T., Ababneh O. H., Al-Bdour M. D. (2013). Ocular manifestations of behçet’s disease in Jordanian patients. *Saudi Journal of Ophthalmology*.

[8] Islam S. M. M., Tabbara K. F. (2002). Causes of uveitis at the eye center in Saudi Arabia: a retrospective review. *Ophthalmic Epidemiology*.

[9] Hamade I. H., Elkum N., Tabbara K. F. (2009). Causes of uveitis at a referral center in Saudi Arabia. *Ocular Immunology and Inflammation*.

[10] Al-Mezaine H. S., Kangave D., Abu El-Asrar A. M. (2010). Patterns of uveitis in patients admitted to a university hospital in riyadh, Saudi Arabia. *Ocular Immunology and Inflammation*.

[11] Al Dhibi H. A., Al Shamsi H. N., Al-Mahmood A. M. (2017). Patterns of uveitis in a tertiary care referral institute in Saudi Arabia. *Ocular Immunology and Inflammation*.

[12] Al Dhahri H., Al Rubaie K., Hemachandran S. (2015). Patterns of uveitis in a university-based tertiary referral center in riyadh, Saudi Arabia. *Ocular Immunology and Inflammation*.

[13] Al-Shakarchi F., Faiz I. (2014). Pattern of uveitis at a referral center in Iraq. *Middle East African Journal of Ophthalmology*.

[14] Ghasemi M., Hosseini S., Shoeibi N., Ebrahimi R. (2018). Patterns of uveitis at a tertiary referral center in northeastern Iran. *Journal of Ophthalmic and Vision Research*.

[15] Bagheri M., Ahoor M.-H., Jafari A., Hashemi H. S., Mohammadkhani M. (2021). Pattern of uveitis in Iran: a systematic review. *Journal of Ophthalmic and Vision Research*.

[16] Cuschieri S. (2019). The STROBE guidelines. *Saudi Journal of Anaesthesia*.

[17] Jabs D. A., Nussenblatt R. B., Rosenbaum J. T. (2005). Standardization of uveitis nomenclature for reporting clinical data. Results of the first international workshop. *American Journal of Ophthalmology*.

[18] Soheilian M., Heidari K., Yazdani S., Shahsavari M., Ahmadieh H., Dehghan M. (2004). Patterns of uveitis in a tertiary eye care center in Iran. *Ocular Immunology and Inflammation*.

[19] Päivönsalo-Hietanen T., Vaahtoranta-Lehtonen H., Tuominen J., Saari K. M. (2009). Uveitis survey at the university eye clinic in turku. *Acta Ophthalmologica*.

[20] Kazokoglu H., Onal S., Tugal-Tutkun I. (2008). Demographic and clinical features of uveitis in tertiary centers in Turkey. *Ophthalmic Epidemiology*.

[21] Tugal-Tutkun I., Onal S., Altan-Yaycioglu R., Huseyin Altunbas H., Urgancioglu M. (2004). Uveitis in behçet disease: an analysis of 880 patients. *American Journal of Ophthalmology*.

[22] BenEzra D., Cohen E., Maftzir G. (2005). Uveitis in children and adolescents. *British Journal of Ophthalmology*.

[23] Khairallah M., Yahia S. B., Ladjimi A. (2007). Pattern of uveitis in a referral centre in Tunisia, north africa. *Eye*.

[24] Rothova A., Buitenhuis H. J., Meenken C. (1992). Uveitis and systemic disease. *British Journal of Ophthalmology*.

[25] Sengün A., Karadağ R., Karakurt A., Sarıcaoğlu M. S., Abdik O., Hasiripi H. (2005). ORIGINAL ARTICLE causes of uveitis in a referral hospital in ankara, Turkey. *Ocular Immunology and Inflammation*.

[26] Biswas J., Narain S., Das D., Sudha K. G. (1996). Pattern of uveitis in a referral uveitis clinic in India. *International Ophthalmology*.

[27] Biswas J., Kharel Sitaula R., Multani P. (2018). Changing uveitis patterns in south India-comparison between two decades. *Indian Journal of Ophthalmology*.

[28] Rahimi M., Mirmansouri G. (2014). Patterns of uveitis at a tertiary referral center in southern Iran. *Journal of Ophthalmic & Vision Research*.

[29] Bertrand P.-J., Jamilloux Y., Ecochard R. (2019). Uveitis: Autoimmunity… and beyond. *Autoimmunity Reviews*.

[30] Abd El Latif E., Nooreldin A., Shikhoun Ahmed M., Elmoddather M., El Gendy W. (2021). Etiology of uveitis in upper Egypt. *Clinical Ophthalmology*.

[31] Nguyen M., Siak J., Chee S.-P., Diem V. Q. H. (2017). The spectrum of uveitis in southern vietnam. *Ocular Immunology and Inflammation*.

[32] Ganesh S. K., Bala A., Biswas J., Ahmed A. S., Kempen J. H. (2016). Pattern of pediatric uveitis seen at a tertiary referral center from India. *Ocular Immunology and Inflammation*.

[33] Engelhard S. B., Bajwa A., Reddy A. (2015). Causes of uveitis in children without juvenile idiopathic arthritis. *Clinical Ophthalmology*.

[34] Çakar Özdal M. P., Yazici A., Tüfek M., Öztürk F., Öztürk F. (2014). Epidemiology of uveitis in a referral hospital in Turkey. *Turkish Journal of Medical Sciences*.

[35] Kianersi F., Mohammadi Z., Ghanbari H., Ghoreyshi S. M., Karimzadeh H., Soheilian M. (2015). Clinical patterns of uveitis in an Iranian tertiary eye-care center. *Ocular Immunology and Inflammation*.

[36] Yang P., Zhang Z., Zhou H. (2005). Clinical patterns and characteristics of uveitis in a tertiary center for uveitis in China. *Current Eye Research*.

[37] Kotake S., Furudate N., Sasamoto Y., Yoshikawa K., Goda C., Matsuda H. (1997). Characteristics of endogenous uveitis in hokkaido, japan. *Graefe’s archive for clinical and experimental ophthalmology*.

[38] Yuen B. G., Tham V. M., Browne E. N. (2015). Association between smoking and uveitis: results from the pacific ocular inflammation study. *Ophthalmology*.

[39] Lin P., Loh A. R., Margolis T. P., Acharya N. R. (2010). Cigarette smoking as a risk factor for uveitis. *Ophthalmology*.

